# Adaptive phenotypic plasticity contributes to divergence between lake and river populations of an East African cichlid fish

**DOI:** 10.1002/ece3.4241

**Published:** 2018-06-27

**Authors:** Jelena Rajkov, Alexandra Anh‐Thu Weber, Walter Salzburger, Bernd Egger

**Affiliations:** ^1^ Zoological Institute University of Basel Basel Switzerland

**Keywords:** adaptive phenotypic plasticity, *Astatotilapia burtoni*, cichlid, lake‐stream, local adaptation, transplant experiment

## Abstract

Adaptive phenotypic plasticity and fixed genotypic differences have long been considered opposing strategies in adaptation. More recently, these mechanisms have been proposed to act complementarily and under certain conditions jointly facilitate evolution, speciation, and even adaptive radiations. Here, we investigate the relative contributions of adaptive phenotypic plasticity *vs*. local adaptation to fitness, using an emerging model system to study early phases of adaptive divergence, the generalist cichlid fish species *Astatotilapia burtoni*. We tested direct fitness consequences of morphological divergence between lake and river populations in nature by performing two transplant experiments in Lake Tanganyika. In the first experiment, we used wild‐caught juvenile lake and river individuals, while in the second experiment, we used F1 crosses between lake and river fish bred in a common garden setup. By tracking the survival and growth of translocated individuals in enclosures in the lake over several weeks, we revealed local adaptation evidenced by faster growth of the wild‐caught resident population in the first experiment. On the other hand, we did not find difference in growth between different types of F1 crosses in the second experiment, suggesting a substantial contribution of adaptive phenotypic plasticity to increased immigrant fitness. Our findings highlight the value of formally comparing fitness of wild‐caught and common garden‐reared individuals and emphasize the necessity of considering adaptive phenotypic plasticity in the study of adaptive divergence.

## 1 INTRODUCTION

Fixed genotypic differences and phenotypic plasticity, that is, the ability of a single genotype to produce different phenotypes depending on the respective environment, have often been viewed as opposing strategies by which organisms can adapt to different environments (Schlichting & Pigliucci, 1998; Kawecki & Ebert, 2004). However, there is growing evidence that under certain conditions, genotypic variability and phenotypic plasticity are complementary mechanisms that jointly facilitate adaptation, speciation and even adaptive radiation (for reviews see: Price, Qvarnstrom, & Irwin, 2003; West‐Eberhard, 2003; Pfennig et al., 2010; Schneider & Meyer, 2017).

In particular, adaptive phenotypic plasticity—the generation of a phenotype that is better suited for a novel environment (Ghalambor, McKay, Carroll, & Reznick, 2007)—can promote the expansion of populations into new niches (Yeh & Price, 2004; Richards, Bossdorf, Muth, Gurevitch, & Pigliucci, 2006; Thibert‐Plante & Hendry, 2011). This is because adaptive phenotypic plasticity can temporarily protect genetic diversity from the direct impact of natural selection, thereby saving time for beneficial mutations to arise and to spread within a population, which may eventually result in genetic differentiation (Schlichting, 2004). Whether adaptive phenotypic plasticity facilitates or constrains adaptive divergence depends on how close the “plastic” phenotype is to the fitness optimum in a given environment.

Theory predicts that if there are no fitness costs associated with plasticity, a close match between the “plastic” phenotype and the fitness optimum would lead to stabilizing selection, so that genetic differentiation is unlikely to build up between populations. On the other hand, any incomplete response relative to a new fitness optimum would lead to directional selection with respect to extreme phenotypes (Price et al., 2003; Ghalambor et al., 2007).

Divergent natural selection between populations exposed to different environments leads to divergence in phenotypic traits that influence survival and reproduction. This adaptive divergence should reduce gene flow between populations because nonadapted migrants will suffer increased costs compared to local residents (Hendry, 2001). To experimentally evaluate whether or not adaptive divergence reduces gene flow in nature, it is necessary to perform manipulative field experiments that mimic secondary contact between divergent populations in a natural habitat (Nosil, 2012). Interestingly, the role of phenotypic plasticity is often overlooked in such experiments, even though selection/introduction experiments in nature are among the most powerful ways to scrutinize the role of plasticity in adaptation (Ghalambor et al., 2007).

Reciprocal transplant experiments provide so far the strongest evidence for divergent selection by demonstrating that ecotypes or incipient species suffer from reduced fitness in each other's environment (reviewed in Hereford, 2009). Such studies are commonly performed in plants (reviewed in Leimu & Fischer, 2008) and are becoming more and more common in insects and fish that inhabit temperate habitats of the northern hemisphere (e.g., Räsänen & Hendry, 2014; Soria‐Carrasco et al., 2014; Gosden, Waller, & Svensson, 2015; Moser, Frey, & Berner, 2016; Soudi, Reinhold, & Engqvist, 2016; Kaufmann, Lenz, Kalbe, Milinski, & Eizaguirre, 2017). Very few such studies have, however, been conducted with animals that inhabit remote areas in the tropical climate (e.g., Thorpe, Reardon, & Malhotra, 2005; Schwartz, Weese, Bentzen, Kinnison, & Hendry, 2010; Bongaerts et al., 2011; Kenkel & Matz, 2016).

Cichlid fishes are one of the most species‐rich vertebrate families, whose natural distribution ranges from Central and South America, across Africa and the Middle East to Madagascar and southern India/Sri Lanka. Cichlids are an important model system in speciation research (Kornfield & Smith, 2000; Kocher, 2004; Seehausen, 2006) and provide well‐described examples of phenotypic plasticity in key ecological traits, such as pharyngeal jaw anatomy, body shape, gill size and brain mass (Greenwood, 1964; Meyer, 1987; Wimberger, 1992; Smits, Witte, & VanVeen, 1996; Bouton, Witte, & Van Alphen, 2002; Crispo & Chapman, 2010; Muschick, Barluenga, Salzburger, & Meyer, 2011). Adaptive phenotypic plasticity in cichlids has been proposed to play a key role in their impressive radiations (Galis & Metz, 1998; Muschick et al., 2011; Schneider & Meyer, 2017).

**Figure 1:**
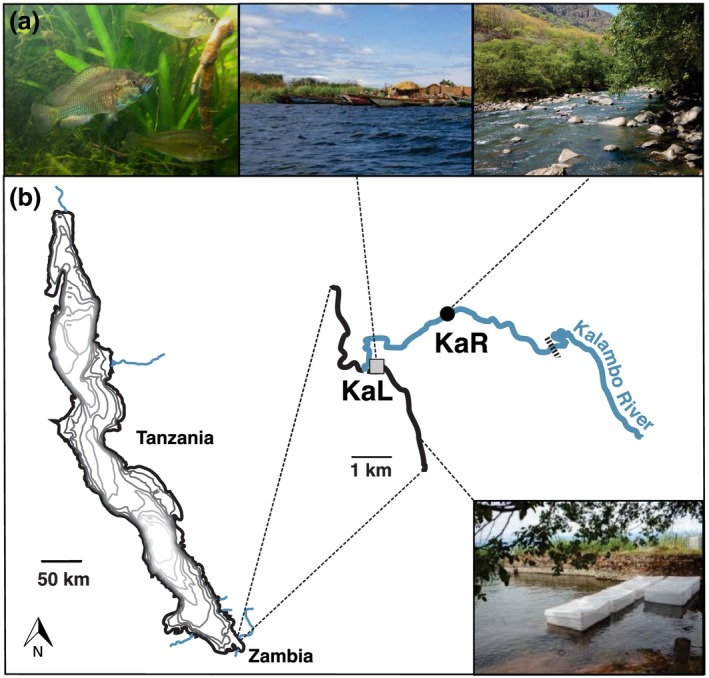
*Astatotilapia burtoni* adult male and two females; lake (KaL—Kalambo Lake) and river (KaR—Kalambo River upstream) habitats (a). Lake Tanganyika with inflowing rivers, location of the experimental enclosures and the two populations used in this study (b)

The Haplochromini represent the most species‐rich and ecologically diverse tribe of African cichlids (Turner, 2007). Among them, *Astatotilapia burtoni* (Günther, 1894) is an excellent model system to study early phases of adaptive divergence. This generalist species inhabits Lake Tanganyika and affluent rivers (Figure 1a). Adjacent lake and river environments differ in both abiotic and biotic conditions including water parameters, habitat structure, prey composition, and parasite communities (Theis, Ronco, Indermaur, Salzburger, & Egger, 2014; J. Rajkov, W. Salzburger, B. Egger, unpublished data). Various lake and river “populations pairs” in *A. burtoni* show similar adaptations to divergent selection regimes despite different levels of genetic differentiation (*F*_ST_) among them (Egger, Roesti, Böhne, Roth, & Salzburger, 2017).

River fish have a shallower body compared to lake fish, which is associated with different flow regimes in the two habitat types, whereas lake fish have a superior mouth position, longer gill rakers as well as more elongated lower pharyngeal jaw bones compared to river fish (Theis et al., 2014). These shifts in trophic structures have been implicated in differential resource use in the two habitat types. Common garden experiments conducted by Theis et al. (2014) demonstrated that differences in body shape and gill raker length have both a plastic and a genetic component and that F1 hybrids are generally intermediate between the parental ecotypes in body shape and gill raker length.

Establishing the link between ecological divergence and fitness differences among populations is crucial to provide evidence that the traits that differ between populations from different habitats are in fact adaptive. In this study, we test the direct fitness consequences of morphological divergence between lake and river *A. burtoni* in nature and evaluate the relative contribution of phenotypic plasticity to fitness and performance (sensu Arnold, 1983). To do so, we performed two independent transplant experiments in Lake Tanganyika, one using wild‐caught juvenile lake and river individuals and a second one using different types of F1 crosses between lake and river fish (pure lake, pure river, and hybrids) that were initially bred in ponds filled with lake water.

Our prediction was that, if adaptation to different environment in *A. burtoni* was mainly due to strong local adaptation, resident lake fish would perform better in lake enclosures than foreign river fish in both of our experiments, and that hybrids between lake and river fish would show an intermediate performance between that of the purebred lines. On the other hand, if there was substantial adaptive phenotypic plasticity, we would not expect a difference in performance of the F1 individuals raised in a common habitat.

## 2 MATERIAL AND METHODS

### 2.1 Study populations and generation of experimental lines

We chose two populations from the Kalambo River system (Figure 1b), a lake population near the estuary (KaL) and an upstream river population (referred to as Ka2 in Theis et al., 2014, 2017; Egger et al., 2017; hereafter referred to as KaR), for the transplant experiments for two main reasons. First, these two populations show the largest difference in body shape and diet composition within any of the lake‐stream population pairs examined by Theis et al. (2014): the lake population (KaL) feeds almost exclusively on plant material and algae, whereas the upstream river population (KaR) feeds mostly on macro‐invertebrates. Second, the facilities where the experiments could be performed in a sheltered bay protected from waves and fishing activities were adjacent (~3 km) to the location where the KaL population was sampled.

For the first transplant experiment, juvenile fish were collected in October 2015 at the two locations (KaL and KaR), using baited minnow traps. For the second transplant experiment, we generated an F1 cohort by crossing wild‐caught adult individuals from the two source populations (KaL and KaR) to create pure lake (KaL × KaL), pure river (KaR × KaR), and hybrid (KaL × KaR) individuals. The parental specimens were caught at the source locations using fishing rods in November 2015. The crosses were raised in concrete ponds supplied with lake water and rocks for shelter between November 2015 and July 2016. Fish were fed with commercial flake food. We used 6–7 ponds for every type of cross, with one male and 3–10 females in each pond to maximize adult and juvenile survival. Hybrid crosses were created in both directions (river female × lake male, river male × lake female). Fish collection in the wild and the transplant experiments were performed under study permits nr. 003376, 004264, 004266 and 004273.

### 2.2 Study design

**Figure 2:**
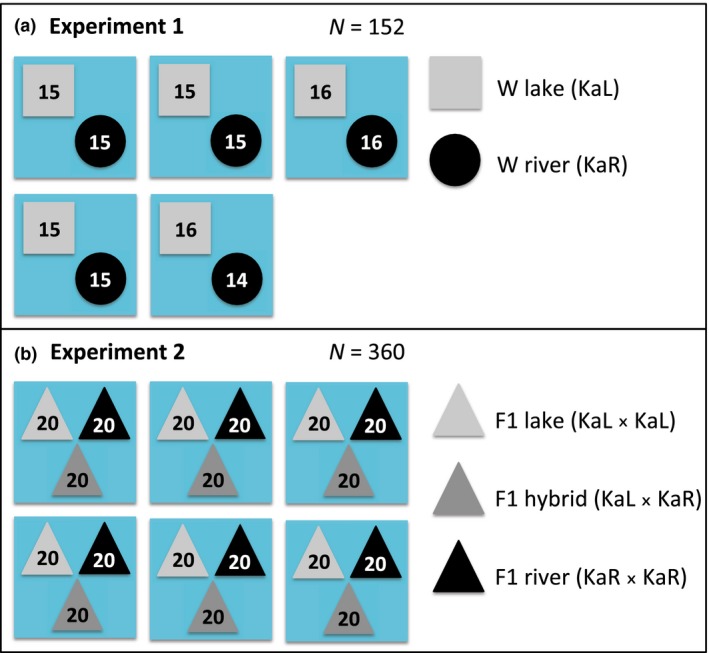
Experimental design of the transplant experiments with sample sizes (blue rectangles indicate enclosures). Experiment 1 with wild‐caught individuals (a), and experiment 2 with F1 crosses raised in ponds with lake water (b). KaL—Kalambo Lake, KaR—Kalambo River upstream

In order to test fitness consequences of the morphological divergence between lake and river *A. burtoni* and to evaluate the relative contribution of phenotypic plasticity to fitness and performance in nature, we performed two transplant experiments, one with wild‐caught juveniles from the two populations (KaL and KaR) and one with juveniles from F1 crosses among and between these populations (KaL × KaL, KaR × KaR, and KaL × KaR) (Figure 2). The aim of the first experiment with wild‐caught individuals was to mimic the natural situation in the case of migration between environments to test for possible immigration barriers. For example, in this experiment individuals could suffer additive parasite infection resulting from early exposure within their habitat and late exposure to parasites after transplant, just as it would occur for natural migrants (Kaufmann et al., 2017). The use of F1 offspring in the second experiment permitted us to assess the effect of plasticity on fitness in a foreign habitat. To this end, we acclimated individuals from both populations for one generation under common conditions (lake water) similar to environmental conditions under which their fitness was going to be measured as advised in Kawecki and Ebert (2004). In both experiments genetic samples from all individuals were taken during all measurements to enable tracking of individual fish using microsatellite genotyping. Experiments were performed in up to six enclosures (Figure 2, S1) positioned in a sheltered bay ~3 km south of the location where KaL population was sampled. The enclosures were 2 m × 2 m × 1 m and positioned in the lake so that they were filled to less than 1 m. They were built in August 2015 using metal poles and 8 mm square mesh, thus allowing the passage of small organisms across the enclosure walls.

In experiment 1, the enclosures were stocked with 30 individuals each at the end of the dry season (low lake water level). In experiment 2, the enclosures were stocked with 60 individuals each during the mid dry season (higher lake water level) (Figure S1).

In this study, we were limited to one‐way transplant experiments in the lake habitat. Although including the reciprocal experimental setup – transplanting lake fish into the river habitat – would have been desirable, this was not feasible given the highly variable river environment and local fishing activities.

### 2.3 Transplant experiment 1: Wild‐caught juvenile performance

Wild‐caught juveniles were photographed with a digital camera (Nikon D5000) on their left side, measured with a ruler (±0.5 mm), weighed on an electronic balance (±5 mg), sexed if possible by visual inspection of external coloration and the genital papilla, fin‐clipped, and tagged with visible implant elastomer tags (VIE, Northwest Marine Technology) before the start of the experiment. Each individual received a population tag (KaL ‐ front left side of the dorsal fin, KaR ‐ front right) to enable subsequent sorting, size matching and counting of recaptured individuals. Experimental fishes were selected for size and sex to achieve a similar size distribution between the two populations within each enclosure and a ~1:1 sex ratio in each population. After this treatment, all the individuals could recover for 24 hr in concrete tanks filled with lake water (one tank per enclosure) to ensure that fish were all in good shape.

Prior to the release of *A. burtoni*, all enclosures were emptied of wild fish and potential predators by angling and extensive minnow trapping and a fine net skirt was sewn to the inside of each cage and buried to prevent fish from escaping. The enclosures were covered on the top with removable 8 mm mesh lids to prevent bird predation. In October 2015 each of the five enclosures used in this experiment was stocked with 15 individuals from the lake (KaL) and 15 from the river (KaR) population, except for enclosure 3, which was stocked with 16 individuals of each type, and enclosure 5, which was stocked with 16 individuals of KaL and 14 of KaR due to handling errors (*n* = 152 total) (Figure 2).

The enclosures were checked twice every day and sampled 15 days post‐release and again after 24 days, which is when the experiment was terminated. We chose this duration because we wanted to terminate the experiment before the individuals were sexually mature and could start reproducing, to prevent the confounding effect of mouthbrooding and egg laying on female weight gain, as well as possible introduction of non‐native populations and hybrid offspring in the wild. To sample the fish in the enclosures, we set 10 minnow traps with inaccessible bait in tea infuser spoons per enclosure one hour before dusk and removed them one hour after dawn on the following day. Recaptured individuals were then assigned to their source population, counted, measured, weighed, sexed if possible, fin‐clipped and a photograph was taken, providing survival information as fitness measure and body mass information as performance measure related to fitness (sensu Arnold, 1983). After the first measurement (15 days post‐release), all fish were set back into their original enclosures. After the second measurement, all recaptured individuals were euthanized with an overdose of clove oil, dissected to confirm their sex, fin‐clipped, and preserved in ethanol.

Genomic DNA from fin clips taken at every time point was extracted using 5% Chelex solution (Casquet, Thebaud, & Gillespie, 2012). The samples were genotyped at five microsatellite loci (Ppun5, Ppun7, Ppun21, UNH130, and Abur82) following the methods described in Theis et al. (2014). Samples from the same individuals taken at different time points were matched using the R package Allelematch (Galpern, Manseau, Hettinga, Smith, & Wilson, 2012) to identify individual fish and to obtain individual‐level data for survival and growth.

### 2.4 Transplant experiment 2: F1 generation and hybrid juvenile performance

All available F1 offspring were pooled per cross type (KaL × KaL, KaR × KaR or KaL × KaR) before the beginning of the experiment, and experimental individuals were selected from that pool with the aim of achieving a similar size distribution between different types of crosses within each enclosure and a ~1:1 sex ratio in each cross type. Selected individuals were tagged (KaL × KaL – front left side of the dorsal fin, KaR × KaR – front right, KaL × KaR – middle right) with the VIE tags. In July 2016, during the mid dry season, each of the six enclosures was stocked with a total of 60 juvenile *A. burtoni* from our F1 line, whereby 20 juvenile individuals were taken from the pure lake cross (KaL × KaL), 20 from the pure river cross (KaR × KaR) and 20 from the hybrid cross (KaL × KaR), resulting in a total number of experimental fish of *n *=* *240 (Figure 2). The densities used in the experiments are close to those observed at the Kalambo lake location where dozens of fish are typically caught in an empty minnow trap within minutes. Fish were measured after 14 and 28 days as described above. Termination of the experiment, including the microsatellite genotyping, was performed as in experiment 1 (see above).

### 2.5 Data analysis

We assessed survival between different experimental populations using generalized linear mixed effect models (GLMMs) with survival as a dependent variable (coded as 0:dead and 1:alive) and population (lake, river, (hybrid)), initial mass, sex (male, female, immature), size deviation (deviation in initial mass from the mean mass per cage), and their interaction (sex: size deviation) as fixed predictors. The replicated enclosures were set as a random effect. The significance of fixed effect parameters was determined by type II χ^2^‐based likelihood‐ratio tests (based on a binomial distribution with logit function; glmer and drop1 function in R).

We calculated absolute growth rates in mg/day and specific growth rates (SGR = 100*(ln (final mass)‐ln (initial mass))/time) for survivors. To correct for individual differences in mass at the beginning of the experiment, specific growth rates were regressed on initial mass. The residual SGR values (rSGR) were used as a measure of relative growth performance (following Scharsack, Kalbe, Harrod, & Rauch, 2007). We assessed growth rates between different experimental populations using linear mixed effect models (LMMs) with growth rate or rSGR as a dependent variable, population (lake, river, (hybrid)), and sex (male, female, immature) as fixed predictors. The replicated enclosures were set as a random effect. The significance of each variable was tested with type II ANOVAs with Kenward‐Roger correction for *F*‐statistics and *df* in linear mixed models (lmer and ANOVA functions in R).

Some individuals were still immature at the end of the experiment, without visible genital papilla or sex‐specific coloration and thus it was not possible to sex them (sexed as immature). We found sex to be the dominant effect in the survival and growth analysis, especially when the immature individuals were included, and therefore, we subsequently conducted the analysis on adults only (immature individuals excluded) and on each sex separately.

Generalized linear mixed effect models and LMMs were calculated with the R package lme4 (Bates, Maechler, Bolker, & Walker, 2015). Significance level for the fixed effects was obtained using the drop1 function of the lme4 package for GLMMs and lmerTest package (Kuznetsova, Brockhoff, & Christensen, 2017) for LMMs. Tukey‐Kramer posthoc tests were applied to test for significance of pairwise comparisons between populations using the lsmeans package (Lenth, 2016). All statistical analyses were performed in R version 3.3.2 (R Core Team, 2016).

## 3 RESULTS

### 3.1 Transplant experiment 1: High overall survival of wild‐caught individuals and faster growth of resident population compared to non‐residents

**Figure 3:**
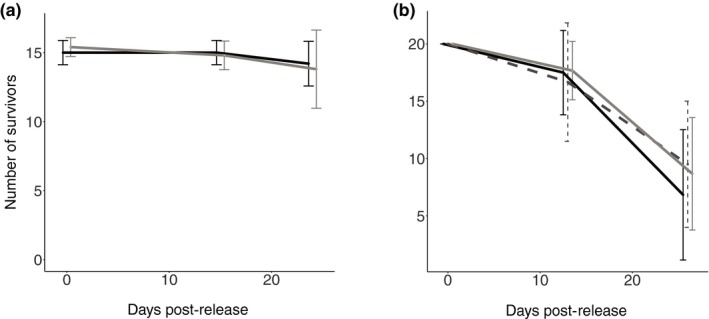
Survival (expressed as the average number of surviving fish ±CI 95%) in the lake habitat for wild‐caught individuals (a) and F1 crosses (b). Lake (light gray), river (black) and hybrid (dark gray dotted) individuals

The survival was high in this experiment (92%) and did not differ between the lake and river fish (population $\chi_{df=1}^{2}$  = 0.340, *p *=* *0.560) (Table 1a, Figure 3a). When only adults were analyzed, there was an effect of size deviation between the experimental fish on survival (size deviation $\chi_{df=1}^{2}$  = 4.513, *p *=* *0.034). When male and females were analyzed separately size deviation only had an effect on male survival (size deviation $\chi_{df=1}^{2}$  = 6.373, *p *=* *0.012).

**Table 1:** Generalized linear mixed models of survival for *A. burtoni* transferred to lake habitat (*df*: degrees of freedom)

	(a) Experiment 1 ‐ wild‐caught fish			(b) Experiment 2 ‐ F1 crosses		
Model 1: whole dataset						
Effect	Residuals *df* = 140			Residuals *df* = 362		
	*df*	χ^2^	*p*	*df*	χ^2^	*p*
Sex	2	1.036	0.596	**2**	**41.910**	**<0.001**
Population	1	0.340	0.560	**2**	**6.034**	**0.049**
Initial mass	1	1.032	0.310	1	0.300	0.584
Size deviation	1	1.169	0.280	1	0.864	0.353
Sex: size deviation	**2**	**9.968**	**0.007**	**2**	**6.761**	**0.034**

Model 2: immature individuals excluded						
Effect	Residuals *df* = 111			Residuals *df* = 318		
	*df*	χ^2^	*p*	*df*	χ^2^	*p*
Sex	1	0.106	0.745	1	0.441	0.507
Population	1	0.243	0.622	2	5.784	0.055
Initial mass	1	0.089	0.765	1	0.371	0.542
Size deviation	**1**	**4.513**	**0.034**	1	1.040	0.308
Sex: size deviation	1	1.650	0.199	1	2.435	0.119

Model 3: males only						
Effect	Residuals *df* = 68			Residuals *df* = 172		
	*df*	χ^2^	*p*	*df*	χ^2^	*p*
Population	1	0.007	0.935	2	2.729	0.256
Initial mass	1	1.803	0.179	1	0.871	0.351
Size deviation	**1**	**6.373**	**0.012**	1	3.131	0.077

Model 4: females only						
Effect	Residuals *df* = 40			Residuals *df* = 142		
	*df*	χ^2^	*p*	*df*	χ^2^	*p*
Population	1	0.018	0.892	2	3.217	0.200
Initial mass	1	0.269	0.604	1	0.094	0.760
Size deviation	1	0.258	0.611	1	0.033	0.856

Experiment 1—wild‐caught fish (a), experiment 2—F1 crosses (b). Significant effects (*p* < 0.05) are highlighted in bold.

**Figure 4:**
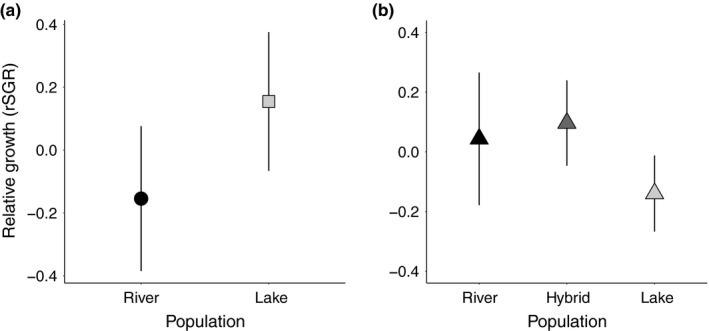
Relative growth performance (rSGR) ±CI 95% in the lake habitat for wild‐caught individuals (a) and F1 crosses (b)

Models with relative (rSGR) (Table 2) and absolute growth rate (Table S1) showed comparable results, so we only discuss the results for the relative growth rate here. Absolute growth rate values are shown in Figure S2. Relative growth rate was associated with sex and population of origin (sex *F*_2,131_ = 12.229, *p *<* *0.001; population *F*_1,130_ = 7.665, *p *=* *0.006) (Table 2a, Figure 4a). When immature individuals were excluded, the effect of sex was comparable to the population effect (sex *F*_1,106.2_ = 5.958, *p *=* *0.016; population *F*_1,106.2_ = 5.739, *p *=* *0.018). Lake fish grew faster than river fish in their local environment, and males grew faster than females. Relative growth rate was higher in the lake males than in river males (population *F*_1,64.8_ = 6.509, *p *=* *0.013), but was not different between the lake and river females (population *F*_1,38.9_ = 0.104, *p *=* *0.749).

**Table 2:** Analyses of variance tables of mixed effect models on relative growth (rSGR)

(a) Experiment 1 ‐ wild‐caught fish					(b) Experiment 2 ‐ F1 crosses			
Model 1: whole dataset								
Effect	num.*df*	den.*df*	*F*	*p*	num.*df*	den.*df*	*F*	*p*
Sex	**2**	**131**	**12.229**	**<0.001**	**2**	**141.3**	**18.418**	**<0.001**
Population	**1**	**130**	**7.665**	**0.006**	2	142.5	1.748	0.178

Model 2: immature individuals excluded								
Effect	num.*df*	den.*df*	*F*	*p*	num.*df*	den.*df*	*F*	*p*
Sex	**1**	**106.2**	**5.958**	**0.016**	**1**	**141.7**	**36.653**	**<0.001**
Population	**1**	**106.1**	**5.739**	**0.018**	2	142.5	1.749	0.178

Model 3: males only								
Effect	num.*df*	den.*df*	F	*p*	num.*df*	den.*df*	*F*	*p*
Population	**1**	**64.8**	**6.509**	**0.013**	2	79.9	1.161	0.319

Model 4: females only								
Effect	num.*df*	den.*df*	F	*p*	num.*df*	den.*df*	*F*	*p*
Population	1	38.9	0.104	0.749	2	58.1	2.837	0.067

*F*‐statistic was corrected with the Kenward–Roger approximation for mixed linear models. Experiment 1—wild‐caught fish (a) and experiment 2—F1 crosses (b). Significant effects (*p *<* *0.05) are highlighted in bold.

### 3.2 Transplant experiment 2: Low overall survival and no growth differences among F1 individuals

Survival was much lower in this experiment (42%) than in the experiment with wild‐caught individuals (Figure 3). The number of survivors per enclosure showed a strong positive correlation with variance in size (standard length at the beginning of the experiment) between individuals within the same enclosure (*r *=* *0.87, *R* ^2^ = 0.7, *p *=* *0.024), meaning that survival was higher in enclosures with more variance in size among individuals. There was a sex effect on survival (sex, $\chi_{df=1}^{2}$  = 41.91, *p *<* *0.001, Table 1b) due to higher mortality of immature individuals. When only adults were analyzed, there was no apparent difference in survival among different crosses (population $\chi_{df=1}^{2}$  = 5.784, *p *=* *0.055), with a tendency of lower survival in river individuals than hybrids (*post hoc* test: KaR x KaR – KaL × KaR, *p *=* *0.074, Figure 3b).

Unlike in experiment 1, there was no difference in rSGR among different types of crosses (population *F*_1,142.5_ = 1.749, *p *=* *0.178, Table 2, Figure 4b), with a tendency of lower rSGR in lake individuals than hybrids (*post hoc* test: KaL × KaL – KaL × KaR, *p *=* *0.092). As in experiment 1, males grew faster than females (sex, *F*_1,141.7_ = 36.653, *p *<* *0.001).

## 4 DISCUSSION

The goal of this study was to test for local adaptation in divergent lake and river populations of a generalist East African cichlid fish. Using two different setups, one with wild‐caught individuals and one with F1 crosses including hybrids raised in a common environment, we were able to examine the contribution of phenotypic plasticity to the adaptation of these populations to different environments. We thus provide the first demonstration of adaptive divergence between lake and river populations of a cichlid species at the level of whole‐organism performance, evidenced by higher growth rates in the wild‐caught resident population compared to nonresident fish in the first experiment. On the other hand, we found a strong contribution of adaptive phenotypic plasticity, evidenced by equal growth rates between different types of F1 crosses in the second experiment. In the following, we discuss the findings of this study in the context of adaptive divergence.

### 4.1 Higher survival of wild‐caught fish and mortality due to male aggression

Survival in experiment 1 using wild‐caught individuals was much higher than in experiment 2 using F1 crosses (92 *vs*. 42%). A likely explanation for this result is the lower density of fish in the first experiment (30 *vs*. 60 individuals per enclosure). Alternatively, fitness of wild‐born fish could generally be higher. A transplant experiment in trout, for example, revealed that wild‐born individuals consistently outperformed both the foreign laboratory‐born groups and their laboratory‐born locally produced counterparts (Westley, Ward, & Fleming, 2012).

Survival in experiment 2 was higher in enclosures with more variance in size among the individuals. A similar observation was recently reported in threespine stickleback fish, in which survival was lower for average‐sized individuals within a cage than for individuals whose initial mass was much larger or smaller than their cage mean (Bolnick & Stutz, 2017).

We further found that male survival in experiment 1 was affected by individual's deviation in size from the mean size per enclosure, indicating that male aggression was the most likely causal factor for mortality. This is further substantiated by the inspection of the deceased individuals that we were able to recover on the water surface during the controls of the enclosures; we found that these fish had injuries, likely from fights with conspecifics. *A. burtoni* males are known to be territorial and highly aggressive toward conspecifics (Fernald & Hirata, 1977; Fernald, 1980), and a size difference of less than 10% body length has been shown to provide a significant advantage to the larger opponent in territorial combats (Alcazar, Hilliard, Becker, Bernaba, & Fernald, 2013).

### 4.2 Higher growth rate in wild‐caught lake fish but not in F1 crosses

As predicted for local adaptation, we found higher growth rates in wild‐caught resident lake individuals in their native environment in experiment 1 compared to river fish. Yet, contrary to our prediction for local adaptation, there was no apparent difference in growth of F1 individuals in experiment 2. The lake‐river hybrids that were expected to show intermediate performance even grew slightly faster than purebred F1 individuals (Figure 4b). This mirrors results from other systems in which the fitness of some hybrid genotypes equals or exceeds that of purebreds (Rundle, 2002). A large body of research on stickleback provides possible explanations for our results. In a recent stickleback study, Best et al. (2017) found that F1 hybrids performed best in a mesocosm experiment and suggested that this might result from increased heterozygosity in hybrids, helping them overcome the effects of mildly deleterious alleles, or from novel combinations of dominant alleles at different loci. However, because F1s tend to be heterotic, and outbreeding depression is often not expressed until the F2 or later generations, conclusions about the relative fitness of hybrids must be tentative (Lexer, Randell, & Rieseberg, 2009). River stickleback, whether migrants or residents, were found to generally grow faster than lake fish (Scharsack et al., 2007; Kaufmann et al., 2017), suggesting a river‐specific trait of faster growth in this species. It has also been suggested that selection on juvenile hybrid stickleback may be weaker than detected in adults (Hatfield & Schluter, 1999). Future studies should aim to investigate fitness of adult hybrids between *A. burtoni* lake and river populations.

In our experiments it was not possible to directly compare the growth rates of wild‐caught river fish and F1 generation raised in lake water due to the different seasons in which the two experiments were performed and due to different densities of fish per enclosure. Growth rates were higher in the first experiment with wild‐caught individuals, which was, however, performed with lower density and at the beginning of the rainy season when water temperature is higher.

### 4.3 Adaptive phenotypic plasticity in *Astatotilapia burtoni*

We found no difference in performance between purebred lake and river crosses in experiment 2 indicating that the juveniles raised in ponds with lake water developed phenotypes with equal fitness as the residents in their non‐native environment.

A common garden experiment that examined plasticity *vs*. genetic contribution for body shape and gill raker length in lake and river *A. burtoni* from the same river (Kalambo) found that F1 offspring from between‐population crosses display intermediate phenotypes in comparison with within‐population crosses (Theis et al., 2014). However, it was also found that the differences between the within‐population crosses raised in the ponds were much smaller than the differences observed between wild types. Moreover, offspring of pure river crosses raised in ponds with lake water was closer to lake fish from the wild than to river fish from the wild with respect to body shape and gill raker length. This indicates that the change in the mean trait values is in the same direction favored by selection in the new environment, but below the new adaptive peak, which is one of the conditions for adaptive phenotypic plasticity to facilitate adaptation (Ghalambor et al., 2007). Theory predicts that at intermediate levels of adaptive plasticity the produced phenotype moves into the attractive domain of the higher fitness peak, and a period of constancy of this new environment leads to a peak shift via “genetic assimilation” (Pigliucci, Murren, & Schlichting, 2006). If the resultant phenotypic variation has a fitness effect, then selection takes place; and if this phenotypic variation has a genetic component, selection leads to ‘‘genetic accommodation,’’ that is, adaptive evolution that involves gene‐frequency change (West‐Eberhard, 2005).

A recent reciprocal transplant experiment in stickleback (Bolnick & Stutz, 2017) detected substantial plastic convergence of immigrant fish toward the gene expression profile of the resident population after translocation (Lohman, Stutz, & Bolnick, 2017). However, stream fish placed in lake cages did not reach the optimum expression in the lake.

Cichlid species that show phenotypic plasticity are often riverine or a part of very recent intralacustrine adaptive radiations (Greenwood, 1964; Meyer, 1989; Smits et al., 1996; Chapman, Galis, & Shinn, 2000) and riverine species show the highest level of adaptive plasticity among the East African cichlids investigated so far, lending support to the ‘flexible stem hypothesis’ (Schneider & Meyer, 2017). If temporal and/or spatial variation is higher in river than in lake habitat, plasticity would be favored over genetic divergence (Scheiner, 1993; Sultan & Spencer, 2002). East African rivers are prone to strong seasonal and interannual fluctuations in water flow rate (Dettinger & Diaz, 2000). Within the Kalambo River, seasonal fluctuations in environmental parameters associated with a seasonal influx of water during the rainy season are supposedly higher than in the lake (Figure S3); thus, *A. burtoni* likely experiences a high degree of temporal and spatial variation in this river system compared to the lake.

High levels of gene flow among populations should favor the evolution or maintenance of phenotypic plasticity over local adaptation (Sultan & Spencer, 2002). Estimated migration rate for the Kalambo River system is higher from the river to the lake (m~2.02E‐04) than *vice versa* (m~6.02E‐05) (Egger et al., 2017), which should favor plasticity in the riverine population. In a study of geographic variation of phenotypic plasticity in another haplochromine cichlid, *Pseudocrenilabrus multicolor*, that inhabits both riverine and swampy areas, high levels of phenotypic plasticity for both gill size and brain mass were observed (Crispo & Chapman, 2010). F1 offspring from populations that are close to the connection between the swamp and river, and thus have the highest potential for dispersal between environments, were shown to have more plastic brains.

Plastic lineages can persist in a new habitat, even if there are no similar niches available, and are therefore expected to have higher potential for adaptive diversification than nonplastic lineages (Schneider & Meyer, 2017). In stickleback, transcriptomic plasticity may play a substantial role in migrants’ adaptation to novel environments (Lohman et al., 2017). In this system genetic divergence and plasticity appear to work together in shaping between‐ecotype differences in gene expression (Lohman et al., 2017) and parallel adaptive phenotypic divergence between lake and stream populations (Oke et al., 2016). Our results provide support for the same forces working together in a cichlid lake‐stream system.

## 5 CONCLUSION

This study provides rare empirical data on fitness estimates in a cichlid species in the wild, using both wild‐caught and F1 individuals. We found a substantial contribution of plasticity to increased immigrant performance in a foreign environment. This finding highlights the value of formally comparing fitness of wild‐caught and common garden‐reared individuals in the study of local adaptation. Given that a single lake‐stream population pair was studied, it is possible that some of the observed patterns are unique to this system. Future studies should aim to overcome logistical challenges and investigate this and other reproductive barriers in additional lake‐stream population pairs including those known to exhibit stronger genomic differentiation in order to achieve a more general understanding of adaptive divergence in this system.

## CONFLICT OF INTERESTS

None declared

## AUTHOR CONTRIBUTION

BE and WS conceived and supervised the study, all coauthors contributed to the experimental design, all authors conducted the fieldwork. JR conducted the molecular laboratory work, analyzed the data, and wrote the manuscript, with feedback from all coauthors.

## DATA ACCESSIBILITY

Individual measurement data available from the Dryad Digital Repository: https://doi.org/10.5061/dryad.7ns4pk2
