# Supplementary material for: Adaptive phenotypic plasticity contributes to divergence between lake and river populations of an East African cichlid fish
Source: Ecol Evol. 2018 Jun 27;8(15):7323–33. doi: 10.1002/ece3.4241 (PMC6106192; doi:10.1002/ece3.4241)
Supplement: Supplementary file 1 [file ECE3-8-7323-s001.docx]

**Supporting information**

Adaptive phenotypic plasticity contributes to divergence between lake and river populations of an East African cichlid fish

Jelena Rajkov^1^, Alexandra Anh-Thu Weber^1^, Walter Salzburger^1^, Bernd Egger^1^

^1^Zoological Institute, University of Basel, Vesalgasse 1, CH-4051 Basel, Switzerland

Email: jelena.rajkov@evobio.eu

(a)

| 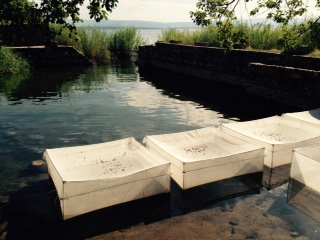 | 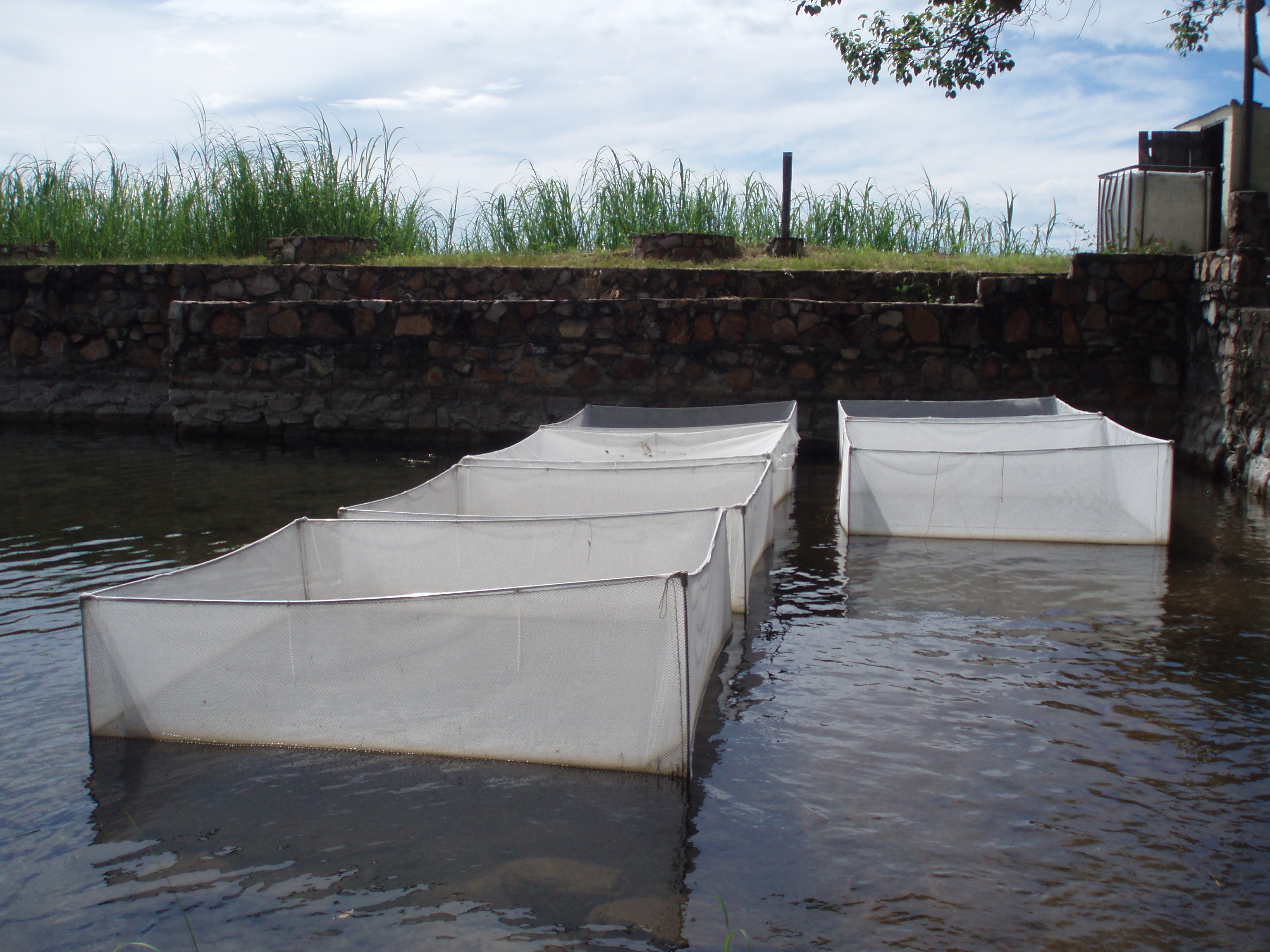 |
| --- | --- |
|  |  |

(b)

| **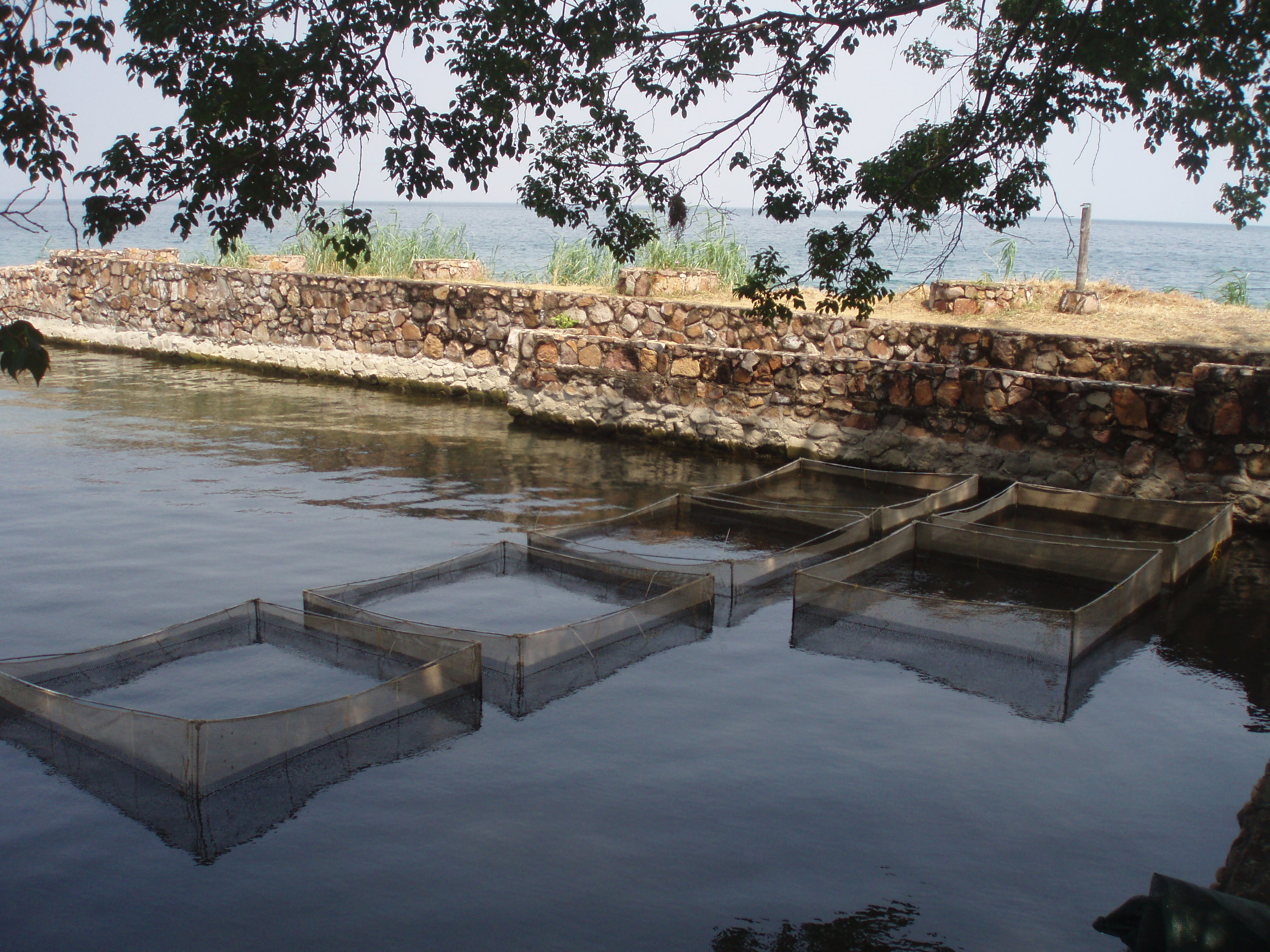** | 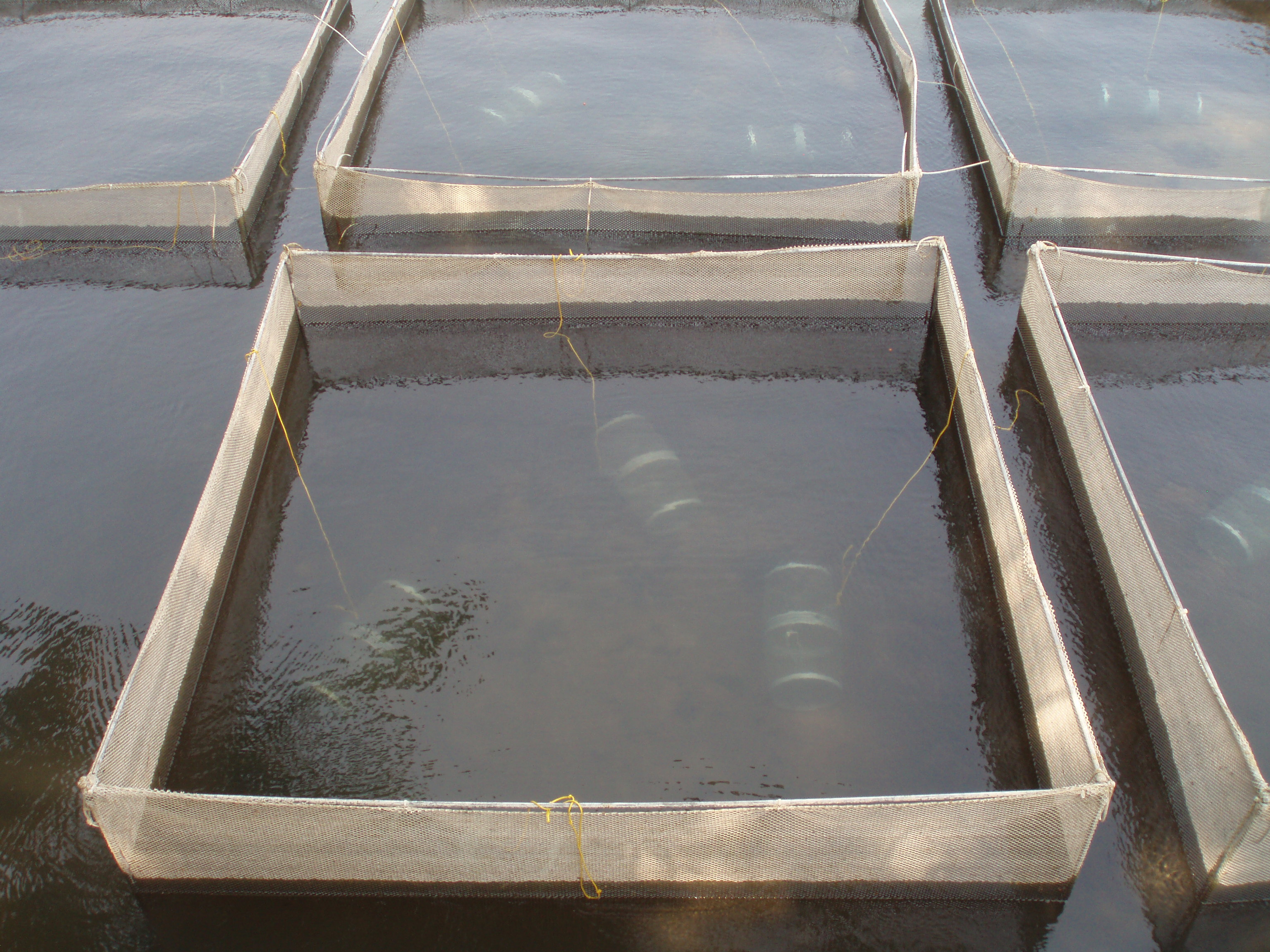 |
| --- | --- |

**Figure S1.** Photographs of enclosures used for the two field transplant experiments in Lake Tanganyika. Experiment 1 conducted during the dry season in 2015 (a), and experiment 2 conducted during the wet season in 2016 (b). For the exact location of the enclosures see Fig. 1 in the main text.

(a) (b)

 ****

**Figure S2.** Growth rates (mg/day) ±CI 95% in the lake habitat for wild-caught individuals (a), and F1 crosses (b).

(a) (b)

| 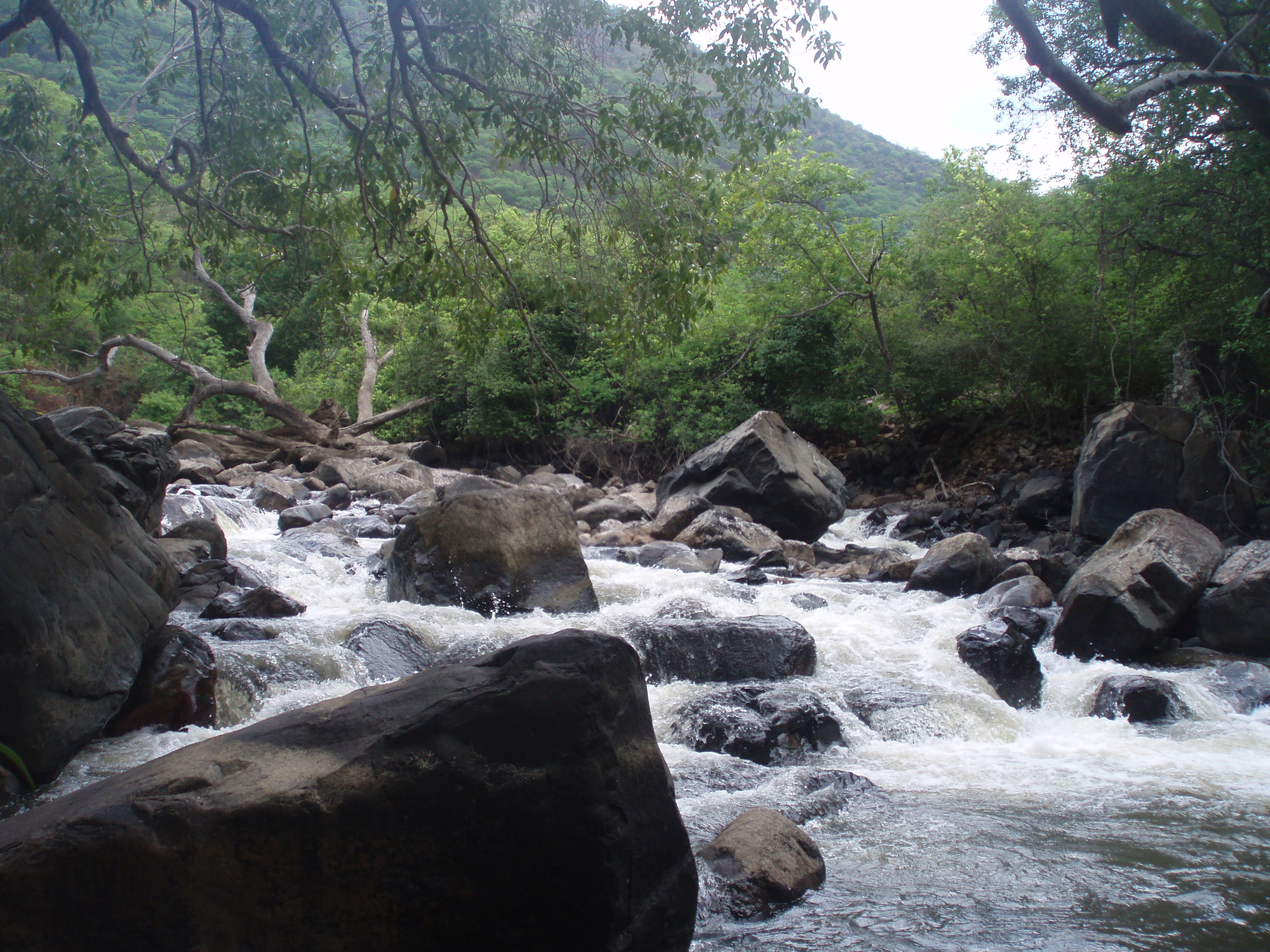 | 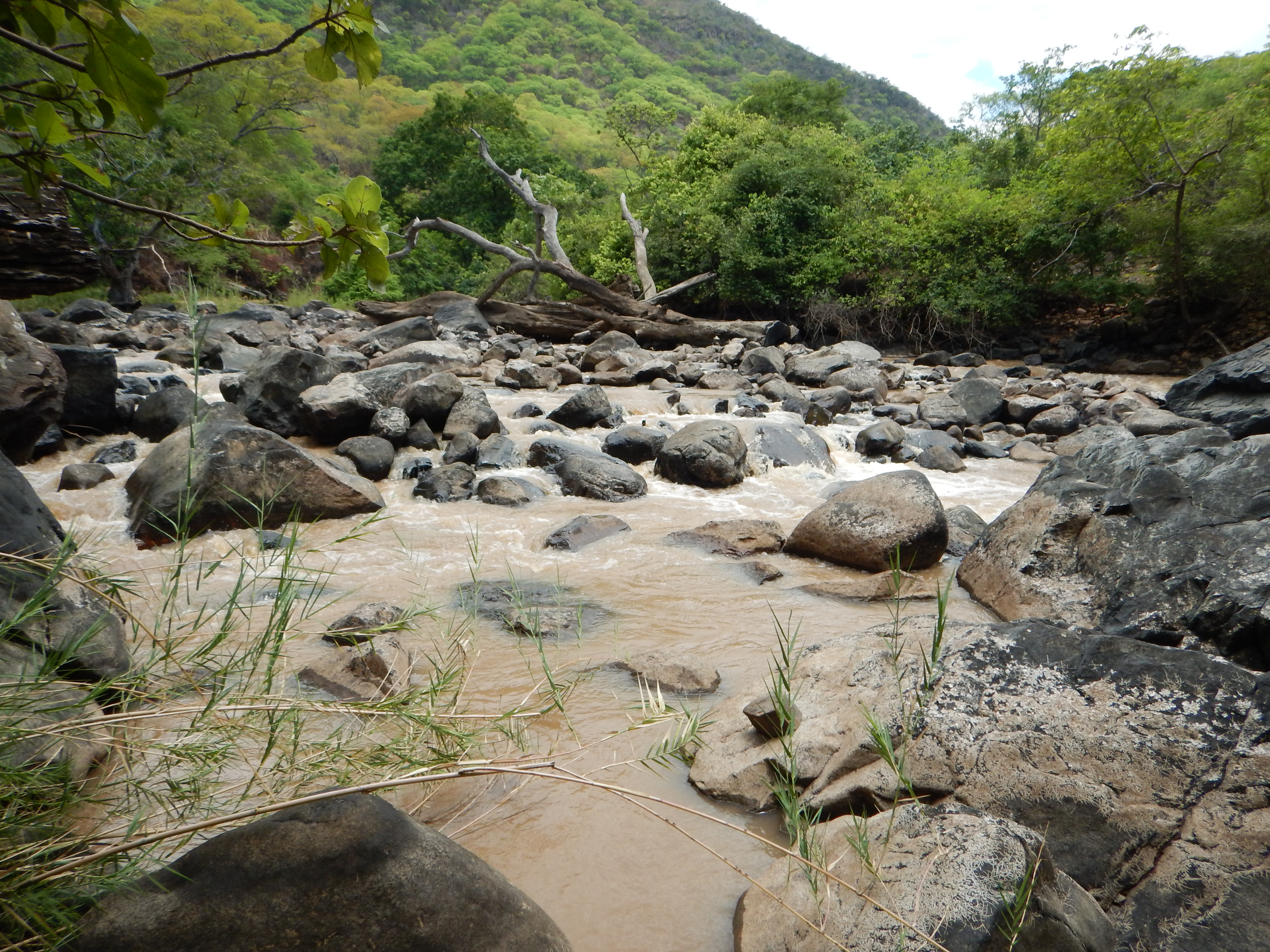 |
| --- | --- |

**Figure S3.** Photographs of Kalambo River upstream (KaR) before (a) and after (b) rain.

**Table S1.** Analyses of variance tables of mixed effect models on growth rate (mg/day). F-statistic was corrected with the Kenward-Roger approximation for mixed linear models. (a) Experiment 1 – wild-caught fish, (b) experiment 2 – F1 crosses. Significant effects (P < 0.05) are highlighted in bold.

|  | (a) Experiment 1 - wild-caught fish | | | |  | (b) Experiment 2 - F1 crosses | | | |
| --- | --- | --- | --- | --- | --- | --- | --- | --- | --- |
|  |  |  |  |  |  |  |  |  |  |
| model 1: whole dataset | | |  |  |  |  |  |  |  |
| Effect | num.d.f. | den.d.f. | *F* | *P* |  | num.d.f. | den.d.f. | *F* | *P* |
| sex | **2** | **132.3** | **19.170** | **<0.001** |  | **2** | **141.2** | **17.872** | **<0.001** |
| population | **1** | **130.1** | **16.330** | **<0.001** |  | 2 | 142.4 | 2.230 | 0.111 |
|  |  |  |  |  |  |  |  |  |  |
| model 2: unknown sex excluded | | | |  |  |  |  |  |  |
| Effect | num.d.f. | den.d.f. | *F* | *P* |  | num.d.f. | den.d.f. | *F* | *P* |
| sex | **1** | **106.5** | **11.037** | **0.001** |  | **1** | **141.6** | **35.492** | **<0.001** |
| population | **1** | **106.2** | **13.246** | **<0.001** |  | 2 | 142.4 | 2.230 | 0.111 |
|  |  |  |  |  |  |  |  |  |  |
| model 3: males only | |  |  |  |  |  |  |  |  |
| Effect | num.d.f. | den.d.f. | *F* | *P* |  | num.d.f. | den.d.f. | *F* | *P* |
| population | **1** | **65.9** | **9.974** | **0.002** |  | 2 | 78.6 | 1.538 | 0.221 |
|  |  |  |  |  |  |  |  |  |  |
| model 4: females only | | |  |  |  |  |  |  |  |
| Effect | num.d.f. | den.d.f. | *F* | *P* |  | num.d.f. | den.d.f. | *F* | *P* |
| population | 1 | 39.4 | 1.517 | 0.225 |  | 2 | 58.2 | 2.003 | 0.144 |
